# Exploring the relationship between delay discounting and physical activity: a meta-analysis of continuous associations

**DOI:** 10.7717/peerj.21343

**Published:** 2026-06-10

**Authors:** Fang Tan, Zhiming Jiao, Kangyuan Guo, Hang Fu

**Affiliations:** 1 School of Medicine and Health Management, Huazhong University of Science and Technology, Wuhan, Hubei, China; 2 The First Affiliated Hospital of Zhengzhou University, Zhengzhou, Henan, China; 3 Institute for Hospital Management of Henan Province, Zhengzhou, Henan, China

**Keywords:** Delay discounting, Physical activity, Exercise, Health behavior

## Abstract

**Background:**

Delay discounting is the reduction in perceived value of rewards over time, a concept studied in psychology and economics to understand decision-making and health behaviors. Its impact on physical activity is gaining interest, but has not been systematically quantified. This meta-analysis aimed to clarify the strength and direction of the continuous association between delay discounting and physical activity.

**Methods:**

Studies incorporating monetary delay discounting and physical activity were identified through PubMed, Web of Science, Embase, and PsycINFO. Random effects meta-analysis was conducted using Pearson’s r as the effect size. Publication bias was assessed using fail-safe N, Begg-Mazumdar, and Egger’s tests, as well as meta-regression and imputation of missing studies.

**Results:**

Twelve effect sizes from 10 studies were included. The primary analysis found a small but significant negative correlation (*r* =  − 0.155, *P* < 0.001) between delay discounting and physical activity. No statistical difference was found between two delay discounting measures (Qbetween = 0.039, *P* = 0.844). Sensitivity analysis confirmed the robustness of the effect size, and no significant bias was detected.

**Conclusions:**

This meta-analysis finds a small negative correlation between delay discounting and physical activity, suggesting that individuals who value the future slightly engage more in physical activity. Further research should explore how delay discounting can be incorporated into exercise interventions, especially for individuals with atypical time preferences, to improve physical activity and health outcomes.

## Introduction

Physical activity (PA) is a pivotal health behavior, offering many benefits beyond the physical realm. Participation in and maintenance of regular exercise leads to significant risk reductions in numerous chronic diseases (Hallal et al., 2006; Moore et al., 2012; Arem et al., 2015; Warburton & Bredin, 2017) and is helpful in weight control (Swift et al., 2014). Beyond these physical benefits, PA also enhances psychological condition (Spence, McGannon & Poon, 2005), bolsters cognitive function (Hillman, Erickson & Kramer, 2008), and elevates quality of life (Langlois et al., 2013; Wu et al., 2017). Interestingly, research suggests that increased levels of physical activity may even positively impact earnings (Kosteas, 2012). Despite the clear benefits of exercise, many people remain inactive. In China, about 62.8% of adults exercise less than three times a week (General Administration of Sport of China, 2022), and in the US, around 53.1% do not meet aerobic exercise guidelines (National Center for Health Statistics, 2022).

The decision to engage in PA often involves a trade-off between immediate costs and long-term benefits. People often prefer immediate or larger rewards, but the decision-making process between smaller-sooner and larger-later rewards is more complex. The value of a reward decreases for people if there is a delay in receiving it, making a larger-later reward potentially less appealing than a smaller-sooner one. This concept, known as delayed discounting (DD) or temporal discounting, describes how rewards lose value over time (Rachlin & Green, 1972; Tesch, Sanfey & Slavin, 2008; Odum, 2011). It is usually measured through choices between smaller-sooner and larger-later rewards (Coller & Williams, 1999; Koffarnus & Bickel, 2014; Bradford, Dolan & Galizzi, 2019). For instance, participants choose between $100 now or $300 in 6 months in an intertemporal choice. The reward or delay is adjusted until both options are equally preferred, known as indifference points. The discount rate parameter k, often modeled hyperbolically, is derived from these points at various delays, a higher k indicates quicker depreciation of future value (Commons et al., 1987). Other parameters available, such as the area under the curve (AUC) (Myerson, Green & Warusawitharana, 2001) and effective delay 50 (Yoon & Higgins, 2008) can also represent the delay discounting rate. While monetary rewards are typical in intertemporal choices, non-monetary commodities like commodities like food, health (Van der Pol & Cairns, 2001; Odum & Rainaud, 2003; Hardisty & Weber, 2009), and other forms of reward can also be considered.

The relationship between DD and PA is often discussion within the framework of intertemporal choice. Many behaviors involve intertemporal choices between current costs and future benefits. Unlike many immediate pleasures, PA is characterized by a conflict between immediate costs (*e.g.*, physical exertion, time commitment, fatigue) and delayed rewards (*e.g.*, weight management, disease prevention, longevity). The decision to engage in PA represents a trade-off between present leisure time and the anticipation of future health gains (Leonard et al., 2013). Consequently, engaging in regular exercise is not merely a single decision but a continuous behavioral chain of choices where an individual must repeatedly prioritize future health benefits over immediate comfort. For individuals with steeper DD rates, the immediate disutility of exercise may outweigh the discounted value of future health gains, presenting a barrier to initiating and maintaining PA habits.

A smaller set of studies suggests that lower DD rate correlates with higher PA levels, indicating that individuals who devalue future rewards less are more likely to engage in PA (Chabris et al., 2008; Daugherty & Brase, 2010; Tate et al., 2015). Furthermore, time discounting has been shown to be associated with physical activity interventions. For example, providing immediate rewards, such as monetary incentives, has been found to be associated with increased participation in PA without significantly reducing the long-term benefits of exercise (Berardi et al., 2024). This suggests that while immediate rewards may shift individuals’ preference for short-term rewards, their promoting effect on PA remains significant. Additionally, another study points out that the time discount rate can moderate the effect of immediate rewards on physical activity, meaning that for those with higher time discount rates, the effect of immediate rewards is more pronounced (Phillips et al., 2020).

Beyond PA, DD has been implicated in a range of health behaviors, including alcohol consumption (Acker et al., 2012; Amlung & MacKillop, 2014) drug use (Arantes et al., 2013; Amlung & MacKillop, 2014), smoking (Ohmura, Takahashi & Kitamura, 2005; Reynolds, 2006), and obesity (Lu et al., 2014; Schiff et al., 2016). For instance, in studies on smoking behavior, time discount has been found to be closely associated with smoking behavior, with higher time discount rates correlating with increased smoking propensity (Kang & Ikeda, 2014).

The role of time discount in physical activity is not only reflected in its impact on individual behavioral decision-making, but also as a potential moderator of interventions. While some meta-analyses have further explored the links between DD and drug addiction (Amlung et al., 2017; Weinsztok et al., 2021), obesity (Amlung et al., 2016; Tang et al., 2019), and other behaviors (Johnson et al., 2021; Strickland et al., 2021), the constant relationship between DD and PA has yet to be thoroughly investigated. This study aims to synthesize existing findings to assess this relationship, with secondary objectives to explore potential moderators.

## Materials & Methods

### Eligibility criteria

To ensure transparency and replicability, this meta-analysis is pre-registered with PROSPERO (#CRD42023404311). Studies were included if they met these criteria: (1) Published, peer-reviewed human research in English. (2) Conducted DD tasks calculating the discount rate k or similar parameters for monetary rewards. (3) Included a PA measurement. (4) Reported correlation coefficients between DD and PA or other variables usable for correlation calculation. (5) Randomized controlled trials (RCTs) and interventional studies were included only if baseline associations were analyzed to avoid the confounding effects of the intervention on the association. Exclusion criteria were defined to eliminate studies that did not meet our analytical needs. Studies were excluded if they met any of the following conditions: (1) reviews, commentaries, conference abstracts, or editorials. (2) Qualitative studies or articles lacking sufficient statistical data (*e.g.*, missing delay discounting rates or extractable correlation coefficients). (3) Studies utilizing non-human subjects. (4) Study designs where the independent association between baseline delay discounting and physical activity could not be isolated due to overlapping interventions.

### Information sources

We systematically searched the following databases from their inception until December 31, 2024: PubMed (includes MEDLINE), Web of Science, Embase (OVID interface, 1974 onwards), and PsycINFO. To ensure comprehensive coverage, we also scanned the reference lists of included studies and relevant reviews identified through our search. The literature search and screening process were conducted between February 3 and February 7, 2025. Data extraction and verification were completed during the same period.

### Search strategy

We developed literature search strategies using the following MeSH terms and text words: (discounting OR intertemporal OR gratification OR time preference) AND (sport OR exercise OR training OR fitness OR physical OR workout). The detailed search formulations for each database are shown in Table S1.

### Data collection process

Two investigators (ZJ and FT) independently screened the search results for titles and abstracts against the inclusion criteria. All studies that appeared to meet the criteria or had any uncertainty were retained for further review. Two other researchers (FT and KG) then independently reviewed the full text to select studies for inclusion. If a consensus could not be reached, a third reviewer (HF) was consulted to make the final decision. The risk of bias for each study was assessed by one reviewer (FT) and verified by another reviewer (KG) based on the Joanna Briggs Institute Critical Appraisal Checklist for analytical cross-sectional study, evaluating aspects such as sample representation, confounding factors, and statistical analysis.

### Data items

Two researchers independently extracted data on the following from each study: lead author, publication year, sample size, participant characteristics, DD task type, DD index, PA measurements, PA type, and effect size (Pearson’s r and Spearman’s Rho (*ρ*)). To avoid within-study interference. We applied the following criteria: (1) Only associations about the most representative variable of interest were considered. Thus, Chan (2017)’s study included only daily exercise time rather than walking time per day, and LeComte, Sofis & Jarmolowicz (2020)’s study included only typical weekly exercise time rather than exercise times in the last week. (2) When studies reported multiple variables, mean effect sizes were calculated for meta-analysis. In Adams & Nettle (2009)’s study, the effect size was the average of two correlation coefficients for moderate and vigorous exercise. The study by Epstein et al. (2021) averaged correlation coefficients for DD with various reward magnitudes as effect sizes. (3) The study by Chabris et al. (2008) comprised three independent studies, all included in the meta-analysis. (4) The Albelwi, Rogers & Kubis (2019)’s study reported DD for different rewards, but only monetary rewards were included. To account for reversed associations in the study by Chabris et al. (2008) and Sukumar et al. (2022) exercise frequency and DD measures were adjusted before analysis.

### Meta-analytic approach

Pearson’s r and Spearman’s Rho (*ρ*) were the primary effect sizes, with *ρ* converted to r and then to Fisher’s Z. Due to significant methodological differences, a random effects model was used, and heterogeneity was assessed with the Q statistic and I^2^ values. A sensitivity analysis was conducted to assess the impact of individual effect sizes by omitting each one and re-estimating the overall effect. The moderator analysis explored differences based on DD measurement using the Q statistic in a fixed-effects model. Five indices were used to evaluate publication bias. The classic fail-safe N indicates how many unpublished studies would nullify the statistical significance (*p* > 0.05) of the aggregate effect, whereas the Orwin fail-safe N estimates the number needed to reduce the effect size to a specific value. Funnel plots of sample size and effect size were analyzed using the two-tailed Begg-Mazumdar test and the one-tailed Egger’s test. Lastly, meta-regression was used to assess the relationship between effect size and publication year. We used Duval and Tweedie’s trim and fill method to adjust effect size estimates with imputed unpublished studies, using Comprehensive Meta-Analysis Version 4.

## Results

### Sample characteristics

**Figure 1:**
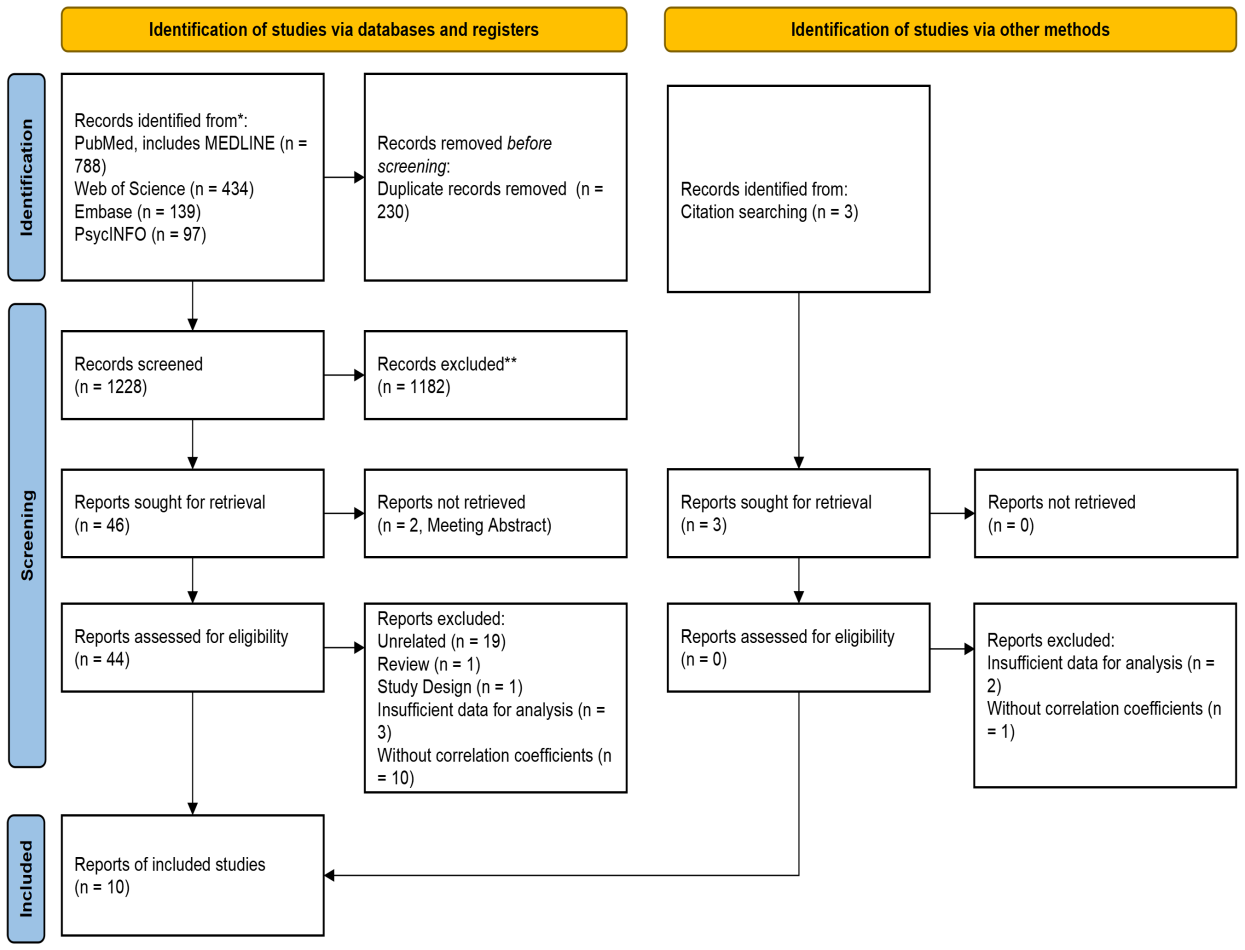
PRISMA flow diagram.

The study selection process is illustrated in the PRISMA flowchart (Page et al., 2021) (Fig. 1). After reviewing the full texts, 10 unique reports met the inclusion criteria and methodological quality standards per the JBI Critical Appraisal Checklist for analytical cross-sectional studies. Overall, the methodological quality of the included studies was generally moderate to high, indicating a low overall risk of bias. Most studies clearly defined the sample inclusion criteria, identified confounding factors, and utilized valid measurement tools for physical activity and delay discounting. Detailed assessment results for each specific criterion are provided in Table S2. This meta-analysis included 10 studies, providing 12 effect sizes, with one report contributing data from three separate studies. Table 1 summarizes the study characteristics. Sample sizes ranged from 12 to 419 participants, totaling 1,782 individuals. Participant ages varied from 20.38 to 65.26 years, with 66.06% being female, and two studies exclusively involved female participants. Eight studies included unrestricted participants, while four targeted specific groups like daily smokers, individuals with normal or overweight status, breast cancer patients, and those with prediabetes. Most studies used questionnaires to assess self-reported exercise behavior, except one that used sensors for actual PA. Seven correlations came from task-based DD measures, and five correlations from the Monetary Choice Questionnaire (MCQ) by Kirby & Marakovic (1996) which uses a 27-item questionnaire to assess DD rates.

**Table 1:** The characteristics of included studies.

ID	Study	Groups	Subgroup	*N*	DD index	Task type	PA measurement	PA variables	Effect size (r)	Age ($\overline{X}$)	Gender (% female)
1	Chabris et al. (2008)	Normalweight and overweight		126	k	MCQ	Questionnaire	Exercise hours/week	−0.228	30.40	50.80%
		College students and community residents		103	k	MCQ	Questionnaire	Exercise hours/week	−0.110	26.10	52.40%
		College graduates		326	k	MCQ	Questionnaire	Exercise hours/week	−0.077	31.40	66.90%
2	Adams & Nettle (2009)	Internet users	Moderate PA	419	log(k)	MDDT	Questionnaire	Days of mod. Exercise/week	−0.086	34.70	81.80%
			Vigorous PA	419	log(k)	MDDT	Questionnaire	Days of vig. Exercise/week	−0.010	34.70	81.80%
3	Tate et al. (2015)	Older adults		137	k	MCQ	YPAS	VAI	−0.273	65.26	60.60%
4	Chan (2017)	College students	Exercise	78	log(k)	MDDT	Questionnaire	Minutes of Exercise/day	0.150	20.38	56.00%
			Walking	78	log(k)	MDDT	Questionnaire	Minutes of walking/day	0.060	20.38	56.00%
5	Sofis, Carrillo & Jarmolowicz (2017)	Female		12	ln(k)	MCQ	IPAQ	Minutes of MVPA/day	−0.017	35.20	100.00%
6	Albelwi, Rogers & Kubis (2019)	Young people	Money DD task	65	k	MDDT	IPAQ	Activity Level by IPAQ	−0.173	24.90	50.00%
			Food DD task	59	k	SDFD	IPAQ	Activity Level by IPAQ	−0.233	24.90	50.00%
			Exercise DD task	67	k	SDET	IPAQ	Activity Level by IPAQ	−0.020	24.90	50.00%
7	Snider et al. (2019)	Daily smokers		303	ln(k)	MDDT	Questionnaire	Questionnaire score	−0.252	39.30	49.20%
8	LeComte, Sofis & Jarmolowicz (2020)	College students	PA: Typically	45	ln(k)	MDDT	Questionnaire	Times of MVPA/week	−0.410	20.83	89.00%
			PA: Last week	45	ln(k)	MDDT	Questionnaire	Times of MVPA in last week	−0.230	20.83	89.00%
9	Epstein et al. (2021)	Prediabetic patients	Reward magnitude:$100	79	log(k)	MDDT	AG	Steps per minute	−0.220	55.20	62.00%
			Reward magnitude:$1000	81	log(k)	MDDT	AG	Steps per minute	−0.270	55.20	62.00%
10	Sukumar et al. (2022)	Female with breast cancer		89	ln(1/k)	MDDT	Questionnaire	Exercise frequency	−0.130	58.70	100.00%

**Notes.**

DD Delay Discounting PA Physical Activity r Pearson’s r k Delay Discounting rate MDDT Money Delay Discount Task IPAQ International Physical Activity Questionnaire SDFD Self-designed Food choice task SDET Self-designed Exercise choice task MCQ Monetary Choice Questionnaire AG Actigraph WGT3X-BT accelerometer MVPA Moderate to Vigorous Physical Activity YPAS Yale Physical Activity Survey VAI Vigorous activity index score

### Meta analysis

**Figure 2:**
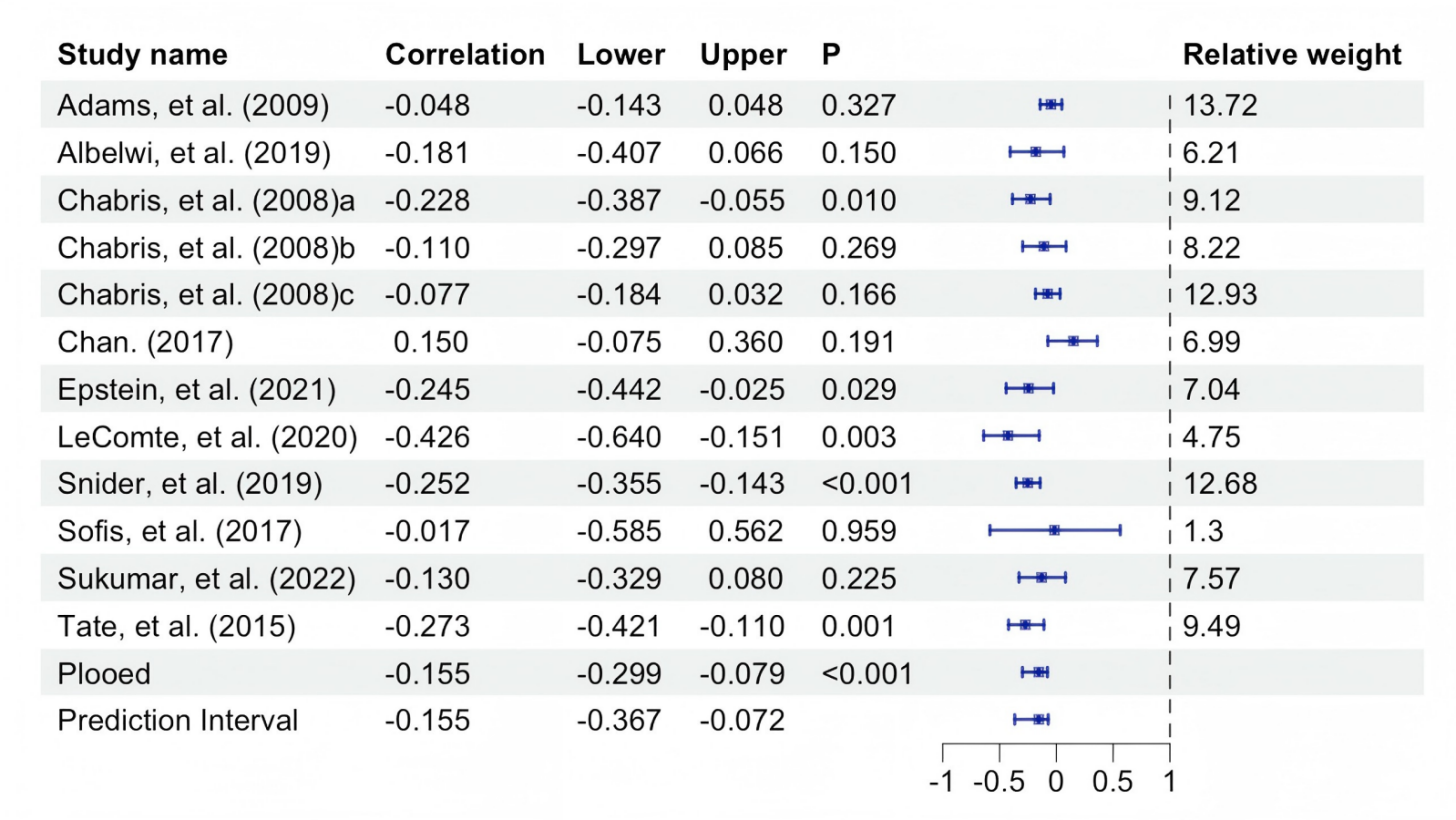
The forest plot of main results.

The meta-analysis incorporated 12 effect sizes, spanning from −0.426 to 0.150, as depicted in Fig. 2. The analysis used a random-effects model, treating the included studies as a random sample from a larger population. The model found a mean effect size (Pearson’s r) of −0.155, with a 95% confidence interval of −0.229 to −0.079. The null hypothesis was rejected (Z = −3.958, *P* < 0.001). Significant heterogeneity was indicated by the Q statistic (*Q* = 24.393, *P* = 0.011), and the I2 statistic showed that 54.90% of the variance was due to actual effects rather than sampling error. Assuming normally distributed true effects (in Fisher’s Z), we estimate a prediction interval of −0.367 to 0.072, indicating the actual effect size in 95% of similar populations falls within this range. Sensitivity analysis (Fig. S1) shows that no single study significantly impacts the mean effect.

### Moderator analyses

Table 2 shows aggregated effect sizes for the examined moderators. No significant difference was found between MCQ and task-based DD measures (Qbetween=0.039, *P* = 0.844), indicating consistent results between the two. However, the task-based DD group exhibited significant heterogeneity (*Q* = 19.229, *P* = 0.004), with 68.80% of the variance due to actual effects rather than sampling error.

### Publication bias

**Figure 3:**
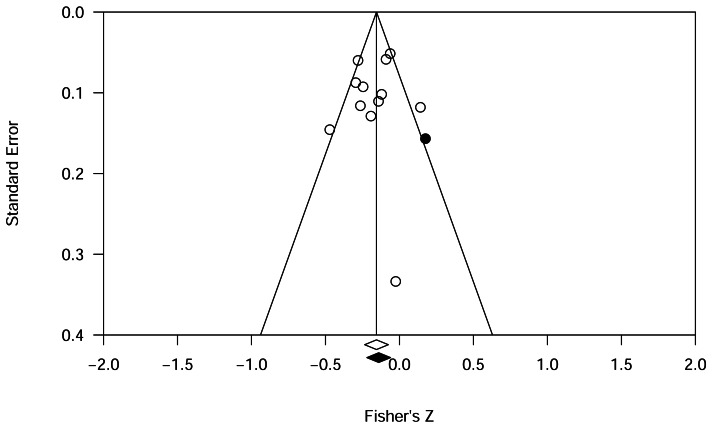
The funnel plot.

The classic fail-safe N was 95, indicating 93 null studies would be needed for the combined 2-tailed *p*-value to exceed 0.050. Orwin’s fail-safe N showed that 11 studies with a mean correlation of 0.000 were needed to reduce the combined correlation to −0.077 (50% of the total effect size). Both the Begg-Mazumdar and Egger’s tests were not significant. Duval and Tweedie’s trim and fill method indicated one missing study on the right side of the mean effect, slightly raising the effect size from −0.155 to −0.140 under the random effects model, showing minimal impact. Visual inspection of funnel plots confirmed that the studies reasonably represented expected effect sizes (See Fig. 3). Meta-regression revealed a small, insignificant association between publication year and effect size, suggesting effect size was independent of publication year.

## Discussion

### Overview of findings

The current meta-analysis synthesized the existing literature on the continuous associations between DD and PA, examined the effects of different DD measurements. Our findings reveal a modest negative link between DD and PA, indicating that those who devalue the future are less likely to engage in PA. The analysis revealed significant variability, but no link was found between DD measure types and this heterogeneity. Task-based DD measures showed more variability compared to MCQ, which had less. Sensitivity analysis confirmed that no single study significantly impacted the overall effect size, and no publication bias was detected.

### Omnibus outcomes

This meta-analysis addresses a previously unexplored area by synthesizing the continuous associations between DD and PA. Our findings contribute to the understanding of how valuing future outcomes affects exercise behavior. Studies typically measure future temporal perspective in two ways: subjective future focus and DD. Subjective future focus uses scales like the Zimbardo Time Perspective Inventory (Zimbardo & Boyd, 2015) and the Consideration of Future Consequences scale (Strathman et al., 1994) to assess general behavioral tendencies. DD, on the other hand, involves making decisions between smaller immediate rewards and larger delayed rewards. While both DD and subjective future focus are associated with health-related behavior, they yield distinct behavioral outcomes due to assessing distinct aspects of future orientation and engaging different cognitive processes. For instance, MacKillop et al. (2006) found that pathological gambling is linked to DD but not to subjective future-focus measures. A meta-analysis by Sweeney & Culcea (2017), which included 18 studies (14 based on subjective future focus and four based on DD) found a modest but significant correlation (*r* = 0.12, *p* < 0.001) between future time perspective and exercise behavior. This correlation aligns with our findings. However, the most striking finding is that the results of both studies demonstrated a positive relationship between future focus and exercise behavior, regardless of the measurement method.

**Table 2:** Moderator analysis by delay discounting measurement type.

Group	k (studies)	Q (within)	df	*P*	I^2^
MCQ	5	5.125	4	0.275	21.94%
task-based DD	7	19.229	6	0.004	68.80%
Between-group (Qbetween)	–	0.039	1	0.844	–
Overall	12	24.393	11	0.011	54.90%

**Notes.**

k number of effect sizes included in each subgroup

Q (within) represents heterogeneity within each subgroup. Qbetween tests differences in effect size between moderator groups. I^2^ indicates the proportion of variance attributable to true heterogeneity rather than sampling error. Random-effects models were applied throughout.

Previous meta-analyses found small but significant associations between DD and various unhealthy behaviors, such as addictive behavior (*r* = 0.14) (Amlung et al., 2017), gambling (*r* = 0.22), food addiction (*r* = 0.12) (Weinsztok et al., 2021), and cannabis use (*r* = 0.082) (Strickland et al., 2021). Additionally, a meta-analysis reported a mean difference in DD of 0.43 between obese/overweight individuals and controls, indicating that steep DD is a notable feature of obesity (Amlung et al., 2016). However, in contrast to these unhealthy behaviors, our study found a weak negative correlation between DD and exercise, indicating that those who value the future are slightly more likely to engage in healthy behaviors.

Theoretically, this negative association aligns with the intertemporal choice framework and Temporal Self-Regulation Theory. Physical activity involves a clear trade-off. It requires immediate costs, such as physical exertion and time commitment. However, it provides delayed rewards, such as disease prevention and improved overall health. Individuals with higher delay discounting rates tend to significantly devalue these distant health benefits. For these individuals, the immediate disutility of exercise outweighs the heavily discounted value of future health gains. This imbalance creates a behavioral barrier to regular physical activity.

The original hypothesis that DD strongly predicts health behavior was not supported, as the correlations between DD and health behaviors were low (*r* < 0.22), with an R^2^ of only 1%. However, Chabris et al. (2008) suggested that DD may better predict aggregated health behaviors rather than individual ones. Given the conflicting evidence, further research is needed to clarify DD’s predictive power on health behaviors.

### Moderator outcomes

Moderator analysis revealed no significant difference in effect size between studies using MCQ and task-based DD measures, consistent with previous meta-analyses on other health behaviors (MacKillop et al., 2011; Amlung et al., 2017; Weinsztok et al., 2021; Strickland et al., 2021). The results suggest that the MCQ maintains measurement precision as a simple measure of DD and enables quicker estimation of delay discount parameters through questionnaire trials (Kirby & Marakovic, 1996; Kirby, Petry & Bickel, 1999; Kirby, 2009). This could potentially replace laborious task-based DD measures if further validated. While there is significant variability among studies using task-based DD measures, the current research could not perform a valid moderator analysis due to an insufficient number of studies in the literature search. Tang et al. (2019)’s meta-analysis revealed that the method for estimating DD rates significantly influences the observed link between DD and obesity. Positive associations were more evident in studies using “best practices”, real rewards, or parametric methods (Tang et al., 2019). Similarly, Strickland et al. (2021) found that the opportunity to give real rewards in DD tasks could strengthen the relationship between DD and cannabis use, while task type and delayed reward magnitude had no significant moderating effect. Regarding reward type, only one meta-analysis explored its impact on the DD-health behavior correlation, finding a larger but statistically insignificant effect size for food rewards compared to monetary rewards. Despite this, the notion that reward type moderates correlations remains theoretically appealing due to varying DD parameter scales in individual studies (Bickel, Odum & Madden, 1999; Petry, 2001; Coffey et al., 2003; Buono et al., 2017). Overall, DD methods show moderate effects, but much remains unknown. Future research detailing DD estimates could clarify if specific DD task methodologies moderate the relationship between discount rates and key variables.

### Implications for public health research and practice

Our findings add to the evidence linking DD and exercise, showing a weak correlation that suggests DD may not strongly predict individual health behaviors. Future research should examine additional factors, like exercise type, social environment, and personal motivation, to enhance DD’s predictive power in exercise interventions (Phillips et al., 2020; Peters & Buechel, 2010). This approach would provide a better understanding of DD’s role in broader behavioral models and its integration into public health strategies.

One potential avenue to explore is how integrating temporal discounting with other psychological constructs, such as self-control or future orientation, might improve intervention outcomes. For example, individuals who have a high DD might also exhibit other traits, like future-oriented cognition, that make it harder for them to engage in healthy behaviors. By combining DD with these other traits, researchers could develop more nuanced models that better predict PA participation.

### Implications for future research

Future research should move from correlational studies to longitudinal designs to better understand how DD affects the initiation and maintenance of PA over time (Leonard et al., 2013; Eberth, Van der Pol & Kemenev, 2022). This could involve exploring causal mechanisms such as emotional regulation, decision-making, or environmental factors.

While monetary rewards have been common in DD tasks, exploring health-related rewards could provide valuable insights. Our study’s slight negative correlation suggests that examining specific health incentives, like weight management or fitness progress, could enhance intervention strategies (Tsukayama & Duckworth, 2010; Strickland, Lile & Stoops, 2017; Albelwi, Rogers & Kubis, 2019).

Future research should also consider the multifaceted nature of DD, including present bias and its interaction with other time preferences (Loewenstein & Prelec, 1992), to better understand their impact on physical activity behavior. Analyzing these aspects across different stages of health behavior change—initiation, consolidation, and relapse prevention—could lead to more tailored and effective intervention strategies. This dynamic approach may help design strategies that adapt to the evolving nature of health behavior over time.

To facilitate literature retrieval for future systematic reviews and meta-analyses, we recommend that researchers uniformly include standardized keywords such as “delay discounting,” “intertemporal choice,” or “time preference” alongside specific behavioral terms like “physical activity” or “exercise adherence” in their future publications.

### Strengths and limitations

This study is the first to systematically review and meta-analyze the continuous relationship between DD and PA. It features a comprehensive literature search, strict inclusion criteria (focusing on hypothetical monetary tasks), a relatively large sample size, and an analysis of various biases in smaller studies. The findings show a consistent but modest link between DD and PA, supported by quantitative evidence. The aggregate effect sizes observed in this review were comparable to the associations between DD and harmful behaviors, complementing the existing literature on DD and preventive behaviors. These results must be considered in the context of the study’s limitations. First, all included studies were cross-sectional, and the meta-analysis focused on correlations, which means causality and directionality between DD and PA cannot be established from these data alone. Second, the limited number of studies and lack of detailed delay discounting tasks restrict our ability to identify significant moderating variables, including the potential moderating role of specific discounting tasks on the relationship between DD and exercise. Finally, the large sample of adults included in this meta-analysis limits the generalizability of these findings to other groups.

## Conclusions

This meta-analysis provides important insights into the relationship between DD and PA. While a small negative correlation was found, suggesting that individuals who value the future slightly engage more in PA, DD’s predictive power remains limited. These findings align with existing research but highlight the complexity of this relationship. Future studies should focus on incorporating both discount rate and present bias, as well as using longitudinal designs to explore the causal mechanisms between DD and PA. Additionally, more tailored intervention strategies considering individual time preferences could enhance PA promotion efforts. Overall, while DD offers valuable insights, further research is needed to develop more integrative models for predicting and influencing health behaviors.

##  Supplemental Information

10.7717/peerj.21343/supp-1 Supplemental Information 1 The detailed search formulations for each database

10.7717/peerj.21343/supp-2 Supplemental Information 2 The results of the methodological quality assessment

10.7717/peerj.21343/supp-3 Supplemental Information 3 The forest plot of sensitivity analysis

10.7717/peerj.21343/supp-4 Supplemental Information 4 PRISMA checklist

## Additional Information and Declarations

### Data Availability

The following information was supplied regarding data availability: This is a systematic review/meta-analysis.
