# Supplementary material for: Exploring the relationship between delay discounting and physical activity: a meta-analysis of continuous associations
Source: PeerJ. 2026 Jun 10;14:e21343. doi: 10.7717/peerj.21343 (PMC13264275; doi:10.7717/peerj.21343)
Supplement: Supplemental Information 1 [file peerj-14-21343-s001.docx]

Table S1 The detailed search formulations for each database

| Database | Search formula | Results |
| --- | --- | --- |
| PubMed | (("discounting"[Title/Abstract] OR "intertemporal"[Title/Abstract] OR "gratification"[Title/Abstract] OR "time preference"[Title/Abstract]) OR ("Delay Discounting"[Mesh])) AND (("sport"[Title/Abstract] OR "exercise"[Title/Abstract] OR "training"[Title/Abstract] OR "fitness"[Title/Abstract] OR "physical"[Title/Abstract] OR "workout"[Title/Abstract]) OR ("Exercise"[Mesh])) | 682 |
| Web of Science | (((((((((((((((((((((((TS=(Discounting, Delay)) OR TS=(Intertemporal Decision-Making)) OR TS=(Decision-Making, Intertemporal)) OR TS=(Intertemporal Decision Making)) OR TS=(Decision Making, Intertemporal)) OR TS=(Temporal Discounting)) OR TS=(Discounting, Temporal)) OR TS=(Intertemporal Preferences)) OR TS=(Intertemporal Preference)) OR TS=(Preference, Intertemporal)) OR TS=(Preferences, Intertemporal)) OR TS=(Delayed Gratification)) OR TS=(Gratification, Delayed)) OR TS=(Deferred Gratification)) OR TS=(Gratification, Deferred)) OR TS=(delay reward discounting)) OR TS=(delayed reward discounting)) OR TS=(delayed rewards discounting)) OR TS=(discounting of delayed rewards)) OR TS=(hyperbolic discounting)) OR TS=(temporal discounting)) OR TS=(temporal reward discounting)) OR TS=(time discounting)) AND (((((((((((((((((((((((((((((((((((TS=(Physical Activity)) OR TS=(Activities, Physical)) OR TS=(Activity, Physical)) OR TS=(Physical Activities)) OR TS=(Exercise, Physical)) OR TS=(Exercises, Physical)) OR TS=(Physical Exercise)) OR TS=(Physical Exercises)) OR TS=(Acute Exercise)) OR TS=(Acute Exercises)) OR TS=(Exercise, Acute)) OR TS=(Exercises, Acute)) OR TS=(Exercise, Isometric)) OR TS=(Exercises, Isometric)) OR TS=(Isometric Exercises)) OR TS=(Isometric Exercise)) OR TS=(Exercise, Aerobic)) OR TS=(Aerobic Exercise)) OR TS=(Aerobic Exercises)) OR TS=(Exercises, Aerobic)) OR TS=(Exercise Training)) OR TS=(Exercise Trainings)) OR TS=(Training, Exercise)) OR TS=(Trainings, Exercise)) OR TS=(biometric exercise)) OR TS=(exercise capacity)) OR TS=(exercise performance)) OR TS=(exercise training)) OR TS=(fitness training)) OR TS=(fitness workout)) OR TS=(physical conditioning, human)) OR TS=(physical effort)) OR TS=(physical exercise)) OR TS=(physical exertion)) OR TS=(physical workout)) and English (Languages) | 383 |
| Embase | ((exercise.ab. or exercise.ti. or sport.ab. or sport.ti. or training.ab. or training.ti. or fitness.ab. or fitness.ti. or physical.ab. or physical.ti. or workout.ab. or workout.ti.) and (discounting.ab. or discounting.ti. or intertemporal.ab. or intertemporal.ti. or gratification.ab. or gratification.ti. or time preference.ab. or time preference.ti.)) to (human and english language and "remove medline records" and embase) | 75 |
| PsycINFO | (TI ( DE "DELAY discounting (Psychology)" OR DE "INTERTEMPORAL choice" OR “Discounting, Delay” OR “Intertemporal Decision-Making” OR “Decision-Making, Intertemporal” OR “Intertemporal Decision Making” OR “Decision Making, Intertemporal” OR “Temporal Discounting” OR “Discounting, Temporal” OR “Intertemporal Preferences” OR “Intertemporal Preference” OR “Preference, Intertemporal” OR “Preferences, Intertemporal” OR “Delayed Gratification” OR “Gratification, Delayed” OR “Deferred Gratification” OR “Gratification, Deferred” OR “delay reward discounting” OR “delayed reward discounting” OR “delayed rewards discounting” OR “discounting of delayed rewards” OR “hyperbolic discounting” OR “temporal discounting” OR “temporal reward discounting” OR “time discounting”) OR AB ( DE "DELAY discounting (Psychology)" OR DE "INTERTEMPORAL choice" OR “Discounting, Delay” OR “Intertemporal Decision-Making” OR “Decision-Making, Intertemporal” OR “Intertemporal Decision Making” OR “Decision Making, Intertemporal” OR “Temporal Discounting” OR “Discounting, Temporal” OR “Intertemporal Preferences” OR “Intertemporal Preference” OR “Preference, Intertemporal” OR “Preferences, Intertemporal” OR “Delayed Gratification” OR “Gratification, Delayed” OR “Deferred Gratification” OR “Gratification, Deferred” OR “delay reward discounting” OR “delayed reward discounting” OR “delayed rewards discounting” OR “discounting of delayed rewards” OR “hyperbolic discounting” OR “temporal discounting” OR “temporal reward discounting” OR “time discounting”)) AND (TI ( DE "PHYSICAL activity" OR DE "EXERCISE" OR DE "PHYSICAL fitness" OR “Physical Activity” OR “Activities, Physical” OR “Activity, Physical” OR “Physical Activities” OR “Exercise, Physical” OR “Exercises, Physical” OR “Physical Exercise” OR “Physical Exercises” OR “Acute Exercise” OR “Acute Exercises” OR “Exercise, Acute” OR “Exercises, Acute” OR “Exercise, Isometric” OR “Exercises, Isometric” OR “Isometric Exercises” OR “Isometric Exercise” OR “Exercise, Aerobic” OR “Aerobic Exercise” OR “Aerobic Exercises” OR “Exercises, Aerobic” OR “Exercise Training” OR “Exercise Trainings” OR “Training, Exercise” OR “Trainings, Exercise” OR “biometric exercise” OR “exercise capacity” OR “exercise performance” OR “exercise training” OR “fitness training” OR “fitness workout” OR “physical conditioning, human” OR “physical effort” OR “physical exercise” OR “physical exertion” OR “physical workout”) OR AB ( DE "PHYSICAL activity" OR DE "EXERCISE" OR DE "PHYSICAL fitness" OR “Physical Activity” OR “Activities, Physical” OR “Activity, Physical” OR “Physical Activities” OR “Exercise, Physical” OR “Exercises, Physical” OR “Physical Exercise” OR “Physical Exercises” OR “Acute Exercise” OR “Acute Exercises” OR “Exercise, Acute” OR “Exercises, Acute” OR “Exercise, Isometric” OR “Exercises, Isometric” OR “Isometric Exercises” OR “Isometric Exercise” OR “Exercise, Aerobic” OR “Aerobic Exercise” OR “Aerobic Exercises” OR “Exercises, Aerobic” OR “Exercise Training” OR “Exercise Trainings” OR “Training, Exercise” OR “Trainings, Exercise” OR “biometric exercise” OR “exercise capacity” OR “exercise performance” OR “exercise training” OR “fitness training” OR “fitness workout” OR “physical conditioning, human” OR “physical effort” OR “physical exercise” OR “physical exertion” OR “physical workout”) ) | 91 |
