# Supplementary material for: Exploring the relationship between delay discounting and physical activity: a meta-analysis of continuous associations
Source: PeerJ. 2026 Jun 10;14:e21343. doi: 10.7717/peerj.21343 (PMC13264275; doi:10.7717/peerj.21343)
Supplement: Supplemental Information 2 [file peerj-14-21343-s002.docx]

Table S2 The results of the methodological quality assessment

| Term | 1 | 2 | 3 | 4 | 5 | 6 | 7 | 8 | Inclusion or not |
| --- | --- | --- | --- | --- | --- | --- | --- | --- | --- |
| Adams, et al. (2009) | no | yes | yes | yes | Not applicable | Not applicable | yes | yes | yes |
| Albelwi, et al. (2019) | no | yes | yes | yes | Not applicable | Not applicable | yes | yes | yes |
| Chabris, et al. (2008)a | no | yes | yes | yes | Not applicable | Not applicable | yes | yes | yes |
| Chabris, et al. (2008)b | no | yes | yes | Unclear | Not applicable | Not applicable | yes | yes | yes |
| Chabris, et al. (2008)c | no | yes | yes | yes | Not applicable | Not applicable | yes | yes | yes |
| Chan. (2017) | yes | yes | yes | Unclear | Not applicable | Not applicable | yes | yes | yes |
| Epstein, et al. (2021) | yes | yes | yes | yes | Not applicable | Not applicable | yes | yes | yes |
| LeComte, et al. (2020) | no | yes | yes | yes | Not applicable | Not applicable | yes | yes | yes |
| Snider, et al. (2019) | yes | yes | yes | yes | Not applicable | Not applicable | yes | yes | yes |
| Sofis, et al. (2017) | no | yes | yes | yes | Not applicable | Not applicable | yes | yes | yes |
| Sukumar, et al. (2022) | yes | yes | yes | yes | Not applicable | Not applicable | yes | yes | yes |
| Tate, et al. (2015) | no | yes | yes | yes | Not applicable | Not applicable | yes | yes | yes |

Note. Methodological quality was assessed using the Joanna Briggs Institute (JBI) Critical Appraisal Checklist for Analytical Cross-Sectional Studies. The numbered columns (1–8) correspond to the following criteria:1.Clear inclusion criteria; 2.Detailed description of study subjects and setting; 3.Valid and reliable measurement of exposure; 4.Objective and standard criteria used for measurement of the condition; 5.Identification of confounding factors; 6.Strategies to deal with confounding factors; 7.Valid and reliable measurement of outcomes; 8.Appropriate statistical analysis. “Yes” indicates criterion met; “No” indicates criterion not met; “Unclear” indicates insufficient information; “Not applicable” indicates the criterion was not relevant to the study design.“Inclusion or not” indicates whether the study met minimum quality standards for inclusion in the meta-analysis.
