# Supplementary figures and images for: Exploring the relationship between delay discounting and physical activity: a meta-analysis of continuous associations
Source: PeerJ. 2026 Jun 10;14:e21343. doi: 10.7717/peerj.21343 (PMC13264275; doi:10.7717/peerj.21343)

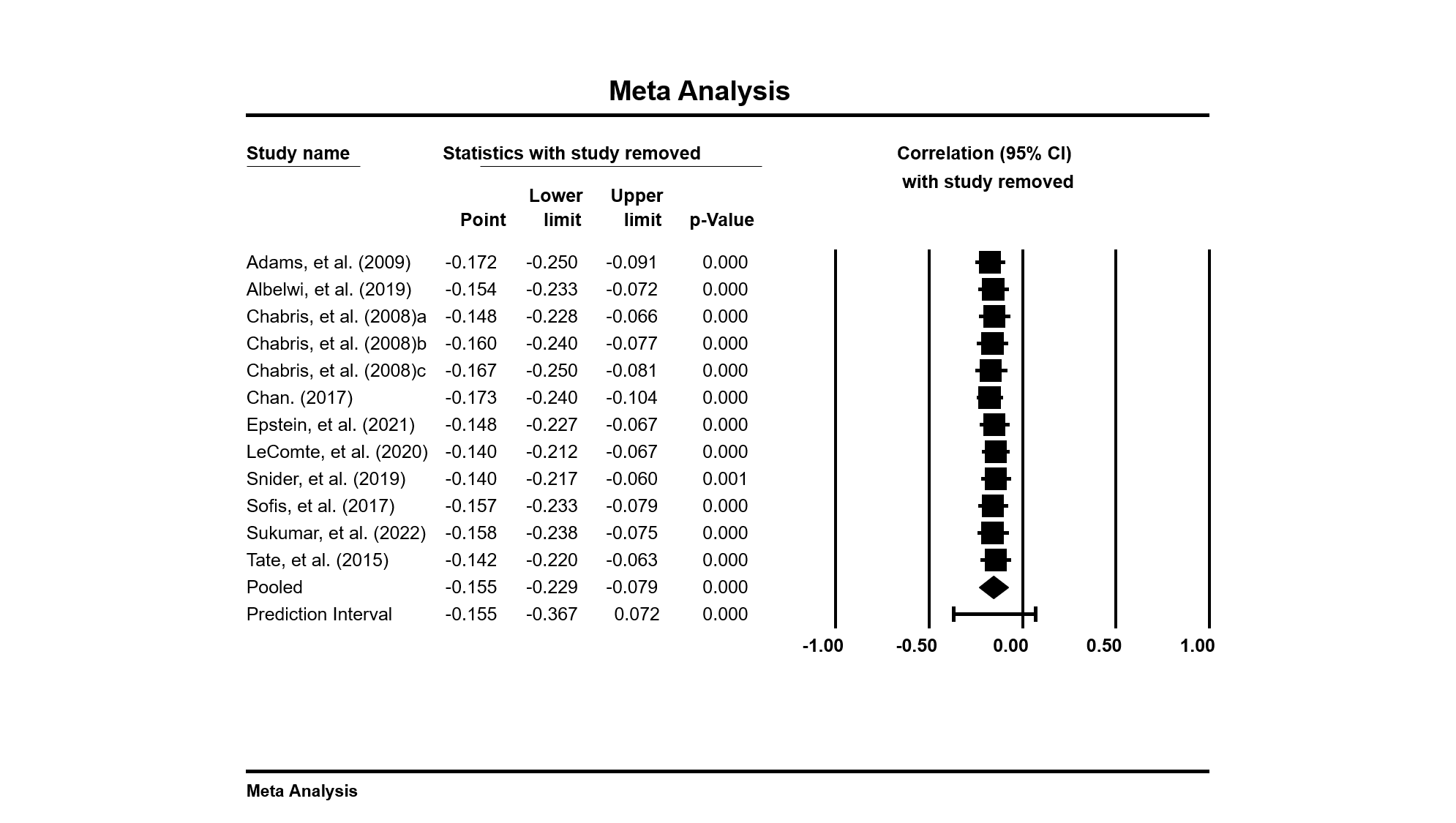

Supplement: Supplemental Information 3 [file peerj-14-21343-s003.png]
